# From By‐Product to Value: Impact of Spent Myrtle Berries on the Quality of Sheep‐Milk Yoghurt

**DOI:** 10.1155/ijfo/1906762

**Published:** 2026-08-18

**Authors:** Angela Carboni, Roberto Cabizza, Pietro Paolo Urgeghe, Francesco Fancello, Massimo Pes, Alessio Silvio Dedola, Severino Zara, Alessandra Del Caro

**Affiliations:** ^1^ Department of Agricultural Sciences, University of Sassari, Sassari, Italy, uniss.it; ^2^ AGRIS Sardegna, Sassari, Italy

**Keywords:** microstructure, myrtle berries powder antioxidant activity, sensory evaluation, texture

## Abstract

In this study, yoghurt from sheep milk was formulated with spent myrtle berry (SMB) powder to enhance its polyphenol content and antioxidant activity. The incorporation of SMB powder into sheep milk yoghurt had a significant effect on its technological properties. Despite the increase in total polyphenols (8.46 mg/100 g FW vs. 1.35 mg/100 g FW in the control) and consequently in the antioxidant activity (1.76 vs. 0.41 in the control), SMB powder caused a substantial alteration of the yoghurt structure, resulting in a reduction in firmness and stickiness of 52.4% and 61% and a threefold increase in syneresis compared to the control, consequently leading to a reduction in gel strength. The analysis of the microstructure revealed a disruption of the protein network that weakened the gel microstructure. Consequently, subsequent studies will be dedicated to the optimisation of the process through the incorporation of thickening agents to enhance consistency and increase the water‐holding capacity. Conversely, the incorporation of SMB into yoghurt did not result in a detrimental impact on sensory quality. The present study is the first to demonstrate the potential of SMB powder as a sustainable functional ingredient in dairy applications.

## 1. Introduction

Milk and dairy products are widely regarded as being among the most effective food categories in terms of generating added value. This can be attributed to their content in essential nutrients, such as proteins, fats, carbohydrates, vitamins A and D, minerals like Ca and P, and prebiotics, as well as their wide availability, which renders them an effective means of valorising agrofood wastes and by‐products, enhancing their nutritional and technofunctional attributes, improving food quality and providing new ingredients for the formulation of innovative dairy foods [1–4]. In recent years, there has been a notable increase in global yoghurt consumption, driven by its functional properties, therapeutic effects and health benefits [4–6]. Recent studies have increasingly focused on the incorporation of powdered by‐products into yoghurt, reflecting a growing trend in the modern food industry [3, 4, 7]. Dietary fibre [8], papaya [9], pineapple peel [10], hazelnut powder [11], apple pomace powder [12], pumpkin peel [7], ground coffee whey powder [13], carrot waste powder [14] and date fibre [15] are some of the waste by‐products used recently to act as health‐promoters and/or exhibit a technological function.

In Sardinia, sheep milk is the most abundant type of milk, with an annual production of approximately 320,000 tons [16], constituting around 68% of Italian sheep milk production and approximately 10% of total European production. Sheep milk is regarded as a ‘functionally active’ food, in which multiple nutritional components coexist, rendering it an excellent ‘food matrix’ [17, 18]. Specifically, the nutritional features that differentiate sheep milk from cow milk include a more favourable fatty acid profile (high conjugated linoleic acid content and low omega‐6/omega‐3 ratio), high protein content, a source of bioactive peptides and a high content of minerals and fat‐soluble vitamins [18]. Sheep milk finds its primary application in the production of traditional cheeses, which are characterised by a prolonged maturation period, with only limited use in the production of fresh dairy products that might preserve and enhance its nutritional characteristics. The current state of knowledge regarding the use of sheep milk in the production of functional dairy products is extremely limited.

Myrtle (*Myrtus communis* L.) is an aromatic medicinal plant indigenous to the coastal regions of the Mediterranean, including North Africa and southern Europe. The berries are characterised by a blue‐black hue, edible, small, round in shape and contain a high number of seeds. In traditional medical systems, the various components of the myrtle plant, particularly the berries, leaves, flowers and essential oils, have been widely utilised as therapeutic agents for the treatment of a range of health complaints, including coughs, gastrointestinal disorders, urinary diseases and skin conditions while also being valued for their antimicrobial activity and wound healing properties [19]. The beneficial properties of the myrtle plant are primarily attributed to the presence of bioactive compounds, including flavonoids, anthocyanins, phenolic acids, lignans, antioxidants, organic acids, fatty acids and minerals, as well as their proximate and nutritional composition in the various parts of the plant [20]. The study of the composition of the myrtle berries is of great importance in Sardinia, where a traditional liqueur called ‘Mirto di Sardegna’ is produced through a process of hydroalcoholic infusion of berries at temperatures above 40°C for a period of 4–6 weeks [21]. Approximately 400 tons of fresh myrtle berries are harvested per year in Sardinia for the production of 2.2 million litres of liqueur [22]. The maceration process gives rise to the generation of a by‐product, spent myrtle berries (SMBs), estimated at approximately 188 tons [22]. The latter is indicative of two distinct phenomena. On one hand, it represents a special waste that requires proper disposal. On the other hand, it is a by‐product with a content of bioactive compounds that can potentially be utilised in the context of a circular economy [23]. The utilisation of SMBs has been predominantly investigated as animal feed, with notable positive impacts on health status, ruminal protein and lipid metabolism and the immune system, along with enhanced milk production and quality [23]. Furthermore, Correddu et al. [23] demonstrated that SMBs contain a notable quantity of polyphenols with high antioxidant capacity, such as flavonols, hydrolysable tannins and phenolic acids, including quinic acid, gallic acid and ellagic acid to which several biological properties have been attributed, including antioxidant, antitumour, antimicrobial and anti‐inflammatory effects [20]. Moreover, recent studies have demonstrated that the considerable volume of by‐products generated during the production of myrtle liqueur can be employed for the extraction of bioactive compounds through mechanical extraction and the utilisation of bio‐based, nontoxic, and biodegradable solvents [23, 24]. To the best of our knowledge, this is the first study to investigate the use of SMBs in a dairy product. The objective of this study, therefore, was to determine the effects of fortification of whole sheep′s milk with SMB powder on the quality of value‐added yoghurt. The effects resulting from the incorporation of SMB were evaluated from multiple perspectives, including chemical–physical, microbiological, rheological, microstructural, textural, and sensory aspects.

## 2. Materials and Methods

### 2.1. SMBs Powder Production

SMBs, the solid by‐product obtained after hydroalcoholic maceration of ripe *M. communis* L. berries for liqueur production, were collected during the 2021–2022 season from a Sardinian distillery (Lucrezio Rau, Berchidda, Sassari, Italy), producer of the traditional ‘Mirto di Sardegna’ liqueur. SMB were then immediately frozen and stored at −20°C until processing. SMB samples were subjected to a drying process at 45°C in an oven (UF260, Memmert GmbH + Co. KG, Schwabach, Germany) for a period of 5 days. Thereafter, the samples were subjected to a subsequent drying step in a vacuum oven (Vacuum Oven 65, Vismara Associate S.p.A., Trezzano, Milan, Italy) at 45°C for a period of 4 days, until a constant weight was achieved. This approach enabled progressive moisture removal at mild temperatures, thereby facilitating the efficient reduction of water activity (a_w_), and ensuring effective preservation of heat‐stable phenolics. In addition, the implementation of a two‐stage drying protocol was deemed to be industrially viable and scalable. Finally, the dried samples were ground into a fine powder using an ultracentrifugal mill (ZM 200, Retsch GmbH, Haan, Germany) with a 0.25 mm sieve and then stored under vacuum (VAC VM 151 HG, Audion Elektro B.V., Weesp, The Netherlands) at −20°C in the dark until analysis.

### 2.2. Characterisation of SMB Powder

#### 2.2.1. Chemical Composition and Water Activity of SMB Powder

The moisture content of the SMB powder was determined by oven‐drying at a temperature of 103^°^C ± 2^°^C until constant weight, in accordance with the ISO 662:2016 [25]. The ash content was determined by incinerating the sample in a muffle furnace at a temperature of 550°C, as specified by AOAC 923.03:2006 [26]. The lipid content was determined gravimetrically by solvent extraction with petroleum ether (Carlo Erba Reagents, Milan, Italy) using an automatic extraction system (SER 158, Velp Scientific, Deer Park, NewYork, United States) in accordance with the AOAC Method 2003.06 [27]. The protein content was estimated from nitrogen (N) determination by elemental analysis (CHN 628, LECO Corporation, St. Joseph, Michigan, United States), following the procedure described by Dahdah et al. [28]. Nitrogen values, expressed as a percentage of the dry weight of the sample (% w/w), were converted to crude protein using the conventional conversion factor of 6.25. The total dietary fibre (TDF) content was determined gravimetrically using the K‐TDFR analytical kit (Megazyme International Ireland Ltd., Bray, Ireland) after enzymatic digestion, following AACC method 32‐05.01 [29] and AOAC Method 985.29 [30]. The CSF6 VELP instrument (VELP Scientifica S.r.l.) was used for the filtration phase. The digestible carbohydrates were estimated by difference, calculated as 100 minus the sum of protein, lipid, TDF and ash contents. Water activity (a_w_) was measured using an electronic hygrometer (Aw‐Win, Rotronic, Bassersdorf, Switzerland) equipped with a Karl‐Fastprobe, calibrated using LiCl standard salt solutions in the range 0.10–0.95, in accordance with ISO 18787:2017 [31]. All measurements were performed in triplicate.

#### 2.2.2. Total Phenolic Content (TPC), Antioxidant Activity (AOA) and Colour of SMB Powder

The extraction of the TPC from SMB powder was performed in accordance with the methodology described by Cannas et al. [32]. In brief, 1 g of powder was subjected to ultrasound‐assisted extraction (UAE) with 20 mL of EtOH/H_2_O 42:58 (v/v) in an ultrasonic bath (DU‐100, ARGO Lab, Carpi, Italy) at 38^°^C ± 2^°^C, 40 kHz and 144 W for 10 min. Subsequently, the mixture was subjected to a centrifugal process at a speed of 5700 rpm for a duration of 10 min at a temperature of 4°C (SL1R Plus, Thermo Scientific, Waltham, Massachusetts, United States). Thereafter, the upper layer of the mixture was filtered using a 0.45 *μ*m cellulose acetate filter (Artiglass, Due Carrare, Italy). The TPC determination was carried out in accordance with the methodology established by Noriega‐Rodriguez et al. [33], with minor modifications. Absorbance was measured at a wavelength of 765 nm, employing a spectrophotometer (Cary 3500, Agilent, Cernusco, Milan, Italy). The data were expressed as grams of gallic acid equivalent (GAE) per 100 g of dry weight (g GAE/100 g DW). All measurements were performed in triplicate.

The AOA was evaluated in accordance with the methodology described by Carboni et al. [34]. Briefly, 2 g of SMB powder were extracted with 20 mL of a methanol/water solution (50:50 v/v) acidified with 37% HCl to a final pH of 2 (Carlo Erba, Milan, Italy), under stirring at room temperature for a period of 1 h. The mixture was then subjected to centrifugation at 4800 rpm for 10 min (SL1R Plus, Thermo Scientific, Waltham, Massachusetts, United States), after which the upper layer of the mixture was collected. The residue was then subjected to a second extraction with 70% (v/v) acetone (Carlo Erba, Milan, Italy) under the same conditions. Subsequently, the two above‐mentioned supernatants were combined and brought to a final volume of 25 mL with methanol, in accordance with the experimental procedure. The AOA was determined by the ABTS assay, following the method reported by Dahdah et al. [35]. The stock solution was then diluted with phosphate‐buffered saline (PBS, 0.1 M, pH 7.4) until reaching an absorbance of 0.70 ± 0.02 at 734 nm, as determined using a UV–VIS spectrophotometer (Cary 3500, Agilent Technologies, Cernusco, Milan, Italy). The results were expressed as *μ*mol of Trolox equivalents per gram of dry weight (*μ*mol TE/g DW). All measurements were performed in triplicate.

The colour of SMB powder was determined using a Minolta CR‐300 colorimeter (Konica Minolta Sensing, Osaka, Japan), equipped with a CR‐300 measuring head. The instrument was calibrated against a white tile that was supplied with the instrument. The measurements were performed in triplicate, with D65 as the illuminant and a standard observer angle of 10°. The colour parameters included L^*^ (lightness), a^*^ (red–green coordinate), b^*^ (yellow–blue coordinate), h° (hue angle) which describes the colour of the powder (from 0° to 360°, where 0° corresponds to red, 90° to yellow, 180° to green and 270° to blue) and C (Chroma) which represents the saturation of the colour.

#### 2.2.3. Microbiological Analyses of SMB Powder

SMB powder was subjected to microbiological analysis to detect potential spoilage and pathogens. The detection of *Listeria monocytogenes* and *Salmonella* spp. was carried out in accordance with ISO 11290‐1/2 and ISO 6579‐1 [36–38], respectively. The enumeration of *Pseudomonas* spp. [39], Enterobacteriaceae [40], *Escherichia coli* [34], presumptive enterococci [41], coagulase‐positive staphylococci [42], aerobic spore‐forming bacteria [43], yeasts and moulds [44] was conducted on selective agar media following procedures described in the literature and previously applied to olive pomace by Carboni et al. [34].

#### 2.2.4. Scanning Electron Microscopy (SEM) Analysis of SMB Powder

The microstructure of the SMB powder was evaluated through the utilisation of SEM. The powder was fixed with 2.5% glutaraldehyde in phosphate buffer (0.1 M pH 7.2) for 24 h. Following this, it was postfixed in 1% of osmium tetroxide (Merk Life Science S.r.l., Milano, Italy) for 1 h. Thereafter, the sample was dehydrated in ascending ethanol concentrations, dried in a critical point drying (CPD) apparatus in a CO_2_ chamber (Jumbo, SPI Supplies West Chester, Pennsylvania, United States), and finally coated with gold/palladium to provide a conductive coating for non‐conductive samples using a sputter coater unit (S150A, Edwards High Vacuum International, Crawley, United Kingdom). The sample was characterised by SEM using a Zeiss EVO LS10 instrument configured in high vacuum mode. Particle size distribution was assessed from SEM micrographs and expressed as equivalent circular diameter (ECD), calculated from particle area measurement. Image processing and particle measurements were carried out using ImageJ software (Version 1.51n, Wayne Rasband, National Institutes of Health, Bethesda, Maryland, United States). Particle size distribution was quantified from ECD measurements by calculating particle size statistics and classifying particles into predefined size intervals (< 1, 1–5, 5–10, 10–50 and > 50 *μ*m).

### 2.3. Yoghurt Production and Analyses

#### 2.3.1. Physicochemical Composition and Somatic Cell Count of Raw Sheep Milk

Whole raw ovine milk was obtained from a local dairy farm (Mangatia, Sassari, Italy) from a flock of healthy Sarda sheep breeds, antibiotic‐free. The fat, protein, casein, lactose and urea content of the milk was determined using the Milkoscan FT + (Foss Electric, Hillerød, Denmark). The measurement of total solids (TSs) was conducted by gravimetric analysis following a drying process to constant weight at 102^°^C ± 2^°^C, in accordance with the ISO method 6731:2010 [45]. The pH measurement was undertaken using a pH metre (Crison Basic 20+, Crison Instruments, Barcelona, Spain), which was equipped with an electrode Lange 50 11 T, Hach Lange GmbH, Vésenaz, Switzerland). The somatic cell count was determined using the flow cytometry method (Fossomatic 5000, Foss Electric, Hillerød, Denmark). Each analysis was performed in duplicate.

#### 2.3.2. Yoghurt‐Making Process

**Figure 1:**
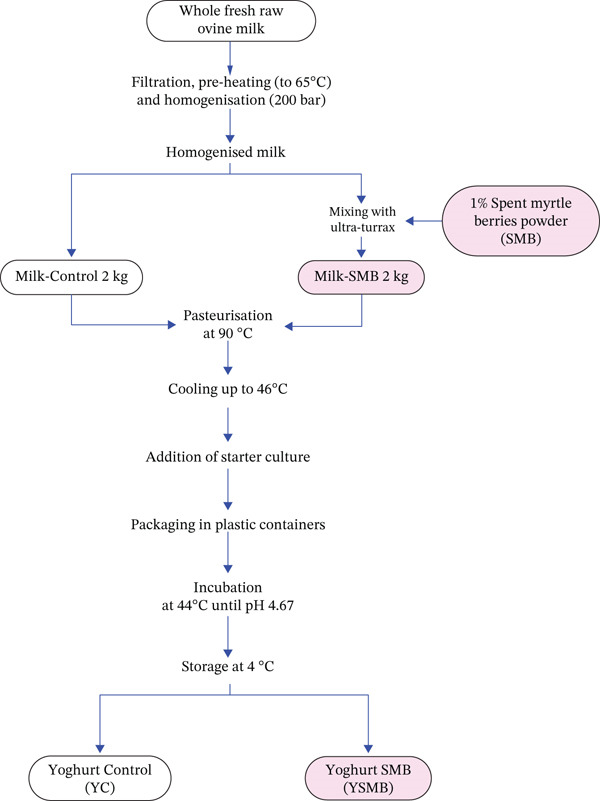
Experimental flowchart of the production of YC and YSMB sheep milk yoghurt.

The yoghurt production procedure is outlined schematically in Figure 1. The selection of the most suitable concentration of SMB powder (1%) to be incorporated into the milk represented the optimal compromise, with the objective being to limit the reduction of gel strength due to the inherent interconnection among polyphenols and caseins, whilst concomitantly ensuring that the bitter and astringent taste perceived at higher concentrations (1.5% and 2%) remained within acceptable limits for consumers. The incorporation of 1% powder thus confirmed the reported concentration ranges for yoghurt supplementation, as evidenced by the extant literature. Indeed, the addition of grape pomace, mulberry pomace and pineapple powder was reported to range from 0.5% to 3%, with 1% being the most prevalent concentration [10, 46, 47].The raw whole milk was subjected to filtration, followed by pre‐heating to 65°C and homogenisation using a two‐stage homogeniser at 200/40 bar (Panther NS3006L, GEA Niro Soavi, Italy). Subsequently, the milk was then divided into two aliquots, with each aliquot being 2 kg. In one aliquot, 1% (10 g/kg of milk) of SMB powder was added immediately, whereas the milk was kept under stirring using a high‐speed dispenser (ULTRA‐TURRAX T25, IKA, Staufen, Germany) (YSMB). The other aliquot was prepared without the powder addition, thus constituting the control batch (YC). Subsequently, both milks were subjected to pasteurisation in a discontinuous method, with temperatures of 90°C for 10 min. The treatment adopted ensures a high degree of whey protein denaturation and promotes whey–casein association, resulting in improved gel structure and water retention, as previously reported in other papers on the thermal behaviour of ovine milk [48, 49]. The milk was subsequently cooled to 46°C, after which the starter culture (0.17 DCU/kg of milk, equivalent to 0.031 g/kg of milk, YO‐MIX 511 LYO 300 DCU, Danisco, Copenhagen, Denmark) was added. Subsequently, the milk was transferred into polypropylene containers (Roll S.r.l., Piove di Sacco, Italy), labelled and incubated at 44°C until a pH of 4.67 was reached. The pH of the yoghurts was continuously monitored and recorded by an 8‐channel transmitter Liquiline CM448 (Endress + Hauser GmbH+Co. KG, Weil am Rhein, Germany) until reaching 4.67. Subsequent to the achievement of the designated pH value, the yoghurts were stored at a temperature of 4°C until the day of analysis.

The pH kinetics of the samples were evaluated by fitting the acidification curves until the targeted pH (4.67), as outlined by Cavallieri and da Cunha [50]. Furthermore, the acidification curve datasets were reduced by the implementation of a sampling frequency of one point every 10 min. The new dataset was then utilised to obtain a first derivative of acidification curves in order to calculate the following parameters: Vm (maximum acidification rate, pH mU/min), Tm (time corresponding to Vm, min), pHm (pH corresponding to Vm), T_50_ (time during which the rate of pH reduction is higher than Vm/2) and pH_50_ (range of the pH during T_50_), as suggested by Spinnler and Corrieu [51].

The production of yoghurt was repeated in triplicate within a limited timeframe (no more than 15 days) to ensure consistency in the raw milk composition and to reduce batch‐to‐batch variations. In order to assess the effect of SMB powder addition to sheep milk intended for yoghurt production and on the structural characteristics of the final product, in addition to acidification monitoring, the following analyses were performed: chemical composition, microbiological analysis, water‐holding capacity (WHC) and syneresis, TPC, AOA, colour, SEM analysis, texture profile analysis, rheological properties and sensory analysis.

#### 2.3.3. Chemical Composition of YC and YSMB Yoghurt Samples

Yoghurt samples were analysed for the following parameters: TS) [52]; fat [53], protein [54]; nitrogen fractions including pH 4.6‐soluble N, 12% trichloroacetic acid soluble N, and 5% phosphotungstic acid‐soluble N, as determined according to Gripon et al. [55]; and total ash [56]. Each analysis was performed in duplicate. The determination of lactose was conducted in accordance with the method reported by Dedola et al. [57]. The yoghurt samples, after deproteinization and derivatisation were analysed using a Varian 3900 gas chromatograph equipped with a split/splitless injector (Varian, Harbor City, California, United States) and a flame ionisation detector (Varian, Harbor City, California, United States). The capillary column used was a Supelco Low Bleed SLBTM—5 ms (Sigma–Aldrich, Milan, Italy), (30 × 0.32 × 0.25 *μ*m). Helium was used as a carrier gas at a flow rate of 1 mL/min; the injector was set at a split ratio of 1:10. Oven temperature was held at 50°C for a duration of 2 min; subsequently, it was elevated to 130°C (heating rate: 10°C/min) and then to 300°C (heating rate: 5°C/min). The temperature was held at 300°C for 5 min, and then it was finally raised to 360°C (heating rate: 30°C/min) and held for an additional 5 min. The temperatures of the injector and detector were established at 300°C. The acquisition and integration of the data was performed using the Star Chromatography Workstation 6.0 software (Varian, Harbor City, California, United States). Peak assignment was performed both on a retention time basis and by spiking with each pure analyte. Each sample was analysed twice.

#### 2.3.4. Microbiological Analyses of YC and YSMB Yoghurt Samples

A microbiological analysis of yoghurts was performed to evaluate the impact of the fortification on the growth of the starter culture employed. In this case, 10 grams of YC and YSMB were collected at 1, 7, 15 and 21 days of storage and aseptically transferred into a stomacher bag containing 90 mL of buffered peptone water (VWR, Milan, Italy). For each batch and sampling time, three technical replicates were analysed. After homogenisation (2 min at room temperature) and preparation of decimal dilutions, 0.1 or 0.5 mL of the appropriate dilutions were plated in duplicate on selective agar media to enumerate the target microorganisms of *Lactobacillus delbrueckii* subsp. *bulgaricus* and *Streptococcus thermophilus*, following the method recently described by Carboni et al. [34], adapted from Dave et al. [58]. Furthermore, at the conclusion of the monitoring period after 21 days of storage, the following microorganisms were identified using distinct standard protocols or different culture media, as previously reported for the SMB powder: *L. monocytogenes*, *Salmonella* spp., *Pseudomonas* spp., Enterobacteriaceae, *E. coli*, presumptive enterococci, coagulase‐positive staphylococci, aerobic spore‐forming bacteria, yeasts and moulds.

#### 2.3.5. WHC and Syneresis of YC and YSMB Yoghurt Samples

The WHC of the yoghurt gel network is indicative of its ability to retain water under conditions of centrifugation stress. This, in turn, is a measure of its structural integrity and resistance to syneresis. The WHC of yoghurt was determined using the following methodology: 25 g of sample was weighed and then subjected to centrifugation at 2500 rpm for 20 min at 4°C (Neya 16 R, Remi Elektrotehnki LTD, Vasai, India) [13]. The WHC percentage was calculated by dividing the difference between the initial weight of the yoghurt and the weight of the released whey by the initial weight of the yoghurt itself. This ratio was then multiplied by 100. Syneresis (%) was determined by the drainage method, in which 30 g of yoghurt sample was placed in a stainless‐steel sieve (800 *μ*m, Endecotts Ltd., London, United Kingdom) and left to drain at room temperature for 15 min [59]. At the conclusion of this period, the weight of whey separated from the gel was calculated as a percentage of the initial weight of the gel and multiplied by 100.

#### 2.3.6. TPC, AOA, Colour and SEM Analyses of YC and YSMB Yoghurt Samples

The extraction of TPC from yoghurt samples was undertaken using the method described by Vázquez et al. [60], with minor adjustments. A total of 8 g of yoghurt were weighed into a 25 mL volumetric flask. Subsequently, 10 mL of a methanol/water (50:50 v/v) solution was added to the flask. Subsequently, 0.5 mL of Carrez I and Carrez II solutions (Supelco, Merck Life Science, Milan, Italy), and 5 mL of acetonitrile (Carlo Erba, Milan, Italy) were added. The flask was filled to volume with a solution of methanol and water (50:50, v/v). Subsequent to each addition, the mixture was vortexed for a period of 1 min (ZX3, Velp Scientifica, Usmate, Italy). The content was transferred to a 50‐mL centrifuge tube, left to rest for 25 min at room temperature, and then subjected to centrifugation at 7800 rpm for 15 min at 5°C. The resulting top layer was collected and transferred to a graduated cylinder for volume measurement. This extract was also utilised in the determination of the AOA. The TPC was determined in accordance with the same procedure that was described for the powder [33]. The results were reported as milligrams of GAEs per 100 g of fresh weight (mg GAE/100 g FW).

The AOA of the yoghurts was assessed using the extract previously prepared for the TPC quantification [60]. Subsequently, the AOA was determined in accordance with the procedure described by Dahdah et al. [35]. The results were expressed as micromolar of Trolox equivalents per gram of fresh weight (*μ*mol TE/g FW).

The colour of the YC and YSMB samples was determined in accordance with the method employed for the SMB powder. Furthermore, the colour difference (*Δ*E) between YSMB and YC was calculated $\left(\Delta \text{E}=\surd \left[{\left({{\text{L}}^{*}}_{\text{YSMB}}-{{\text{L}}^{*}}_{\text{YC}}\right)}^{2}+{\left({{\text{a}}^{*}}_{\text{YSMB}}-{{\text{a}}^{*}}_{\text{YC}}\right)}^{2}\right.\right.\left.\left.+{\left({{\text{b}}^{*}}_{\text{YSMB}}-{{\text{b}}^{*}}_{\text{YC}}\right)}^{2}\right]\right)$ in order to highlight potential variations.

SEM analysis was performed on YC and YSMB samples following the same procedure as for the SMB powder.

#### 2.3.7. Texture Analysis of YC and YSMB Yoghurt Samples

A penetration test was performed on yoghurt samples at a temperature of 4°C by using a TA.XT plus texture analyser (Stable Micro System, Godalming, United Kingdom) equipped with a P‐25 probe (25 mm Cyl. Aluminium, 11305, Stable Micro System) and a 5 kg load cell. The samples, contained in a plastic cup (Roll S.r.l, Piove di Sacco, Italy), were penetrated vertically to a depth of 20 mm at a speed of 1 mm/s and returned to their initial position at the same speed. The maximum force required to break the gel was used as a measure of the firmness (N) of the yoghurt. Furthermore, the stickiness (N), work of shear (N·s), and work of adhesion (N·s) were measured.

#### 2.3.8. Rheological Analyses of YC and YSMB Yoghurt Samples

The steady shear and frequency sweep tests were performed on yoghurt samples using a rheometer (MCR‐92, Anton Paar, Graz, Austria) with a plate‐to‐plate system (diameter: 50 mm and gap: 0.5 mm) at 25°C, as reported by Yeung et al. [61]. The steady shear analysis was conducted at a shear rate of 1–1000 s^−1^, after which the flow behaviour index, consistency index, Casson yield stress and apparent viscosity at 50 s^−1^ were obtained. The viscoelastic properties of each yoghurt sample were obtained by analysing the dynamic shear rheological properties by means of frequency sweep test (G′ = storage modulus; G″ = loss modulus; tan *δ*; complex viscosity at 1 Hz) [61].

#### 2.3.9. Sensory Evaluation of YC and YSMB Yoghurt Samples

YC and YSMB were subjected to an acceptance test, which was conducted at the Department of Agriculture′s sensory analysis laboratory at the University of Sassari. Prior to the evaluation, written consent was requested from the participants. Participants suffering from myrtle allergy and lactose intolerance were excluded from the study. The test was conducted with 100 consumers aged 19–65 years. The evaluation of the YC and YSMB yoghurts was conducted using a scale ranging from 1 to 9, with 1 representing a strong dislike and 9 representing a strong like. The following attributes were evaluated: texture, flavour and overall pleasantness [62]. YC and YSMB samples, identified with a random three‐digit code were presented to the judges in a randomised and balanced order. Water was presented for palate cleansing. The Smart Sensory Box software (Smart Sensory Solutions S.r.l, Sassari) was used to perform the test.

### 2.4. Statistical Analysis

The statistical software Statistica 12.0 (StatSoft, Inc., Tulsa, Oklahoma, United States) was used to perform one‐way analysis of variance (ANOVA) on the measured data. The means were separated using Fisher′s LSD test (95% confidence interval). Bacterial counts were log_10_‐transformed before statistical analysis. The impact of storage time (1, 7, 14 and 21 days) and yoghurt formulation (YC and YSMB) were evaluated through a two‐way analysis of variance (ANOVA) using SPSS Statistics software (Version 22, IBM Corp., Armonk, New York, United States). When significant differences were detected (*p* < 0.05), mean separation was conducted employing Tukey′s post hoc test. The sensory data were analysed using XLSTAT for Windows (Version 2020.1.2, Addinsoft, Paris, France). The analysis of overall liking scores was conducted using a *t*‐test (*p* < 0.05).

## 3. Results and Discussion

### 3.1. Proximate Composition, TPC, AOA, Colour, Microbiological and SEM Analyses of SMB Powder

SMB powder was characterised by a high total fibre content (82.94%), TSs (90.55%) and proteins (7.73%), which were found to be very similar to the content of berry pomace of different species, such as cranberry and lingonberry, which ranged from 50% to 74% on dry weight basis [63, 64] (Table 1). Furthermore, the powder exhibited a high content of TPC (approximately 4 g/100 g on a dry weight basis), as previously reported in the literature [65]. In consideration of the well‐established phenolic profile reported by Correddu et al. [23], the high AOA of the powder can be attributed to its high ellagic acid content, a compound recognised for its antioxidant, antimicrobial and anti‐inflammatory properties. Therefore, it was essential to assess the viability of yoghurt starter bacteria in light of its established antimicrobial properties. The analysis of pathogens and spoilage microorganisms, conducted on three replicate samples of SMB powder, revealed concentrations below the detection limit. The absence of pathogens, in addition to the effects of processing treatments applied during powder production (e.g., drying), may also be ascribed to the intrinsic antimicrobial properties of SMBs, which are mainly associated with their high concentrations of flavonoids, anthocyanins and phenolic compounds [66–68]. These findings suggest that the by‐product is microbiologically safe for use in ovine yoghurt production. In conclusion, the powder exhibited a red‐purple colour with slight brightness.

**Table 1:** Proximal composition, TPC, AOA and colour of SMB powder.

Parameters	^a^Values
Moisture (g/100 g)	9.45 ± 0.45
TS (g/100 g)	90.55 ± 0.45
Ash (g/100 g DW)	3.04 ± 0.01
Lipids (g/100 g DW)	2.94 ± 0.34
Total dietary fibre (g/100 g DW)	80.91 ± 0.61
Protein (g/100 g DW)	7.73 ± 0.10
Water activity (a_w_)	0.41 ± 0.01
TPC (g GAE/100 g DW)	3.68 ± 0.27
AOA (*μ*mol TE/g DW)	293.06 ± 5.96
L^*^	38.07 ± 0.04
a^*^	5.39 ± 0.12
b^*^	10.70 ± 0.01
Chroma	11.97 ± 0.04
Hue	63.3 ± 0.74

Abbreviations: DW, dry weight; TS, total solid; TPC, total phenolic content; AOA, antioxidant activity.

^a^Values represent the mean ± standard deviation (*n* = 3).

**Figure 2:**
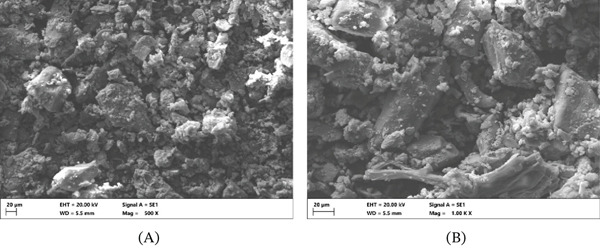
Scanning electron microscopy pictures of SMB powder, at different scale bars and magnifications: (A) 20 *μ*m–500x and (B) 20 *μ*m–1.00 K x.

SEM analysis was undertaken on the SMB powder to ascertain the nature of its surface. The results of the analysis revealed a rough, loose and porous surface, characterised by a notable presence of irregular particles derived from milled berry seeds and plant cell walls (Figure 2). These particles have likely collapsed during the drying process [68]. The particle size analysis demonstrated that SMB exhibited substantial heterogeneity in terms of size distribution, with a mean ECD of 5 *μ*m, and values ranging from 0.9 to 114 *μ*m. The majority of particles fell within the 1–5 *μ*m range (59.8%), followed by particles between 5–10 *μ*m (13.9%) and those smaller than 1 *μ*m (15.8%). A negligible proportion of the particles exceeded 10 *μ*m, with approximately 9.9% falling within the 10–50 *μ*m range and less than 1% exceeding 50 *μ*m.

### 3.2. Milk Chemical Composition

The chemical composition of the milk was as follows: pH 6.60 ± 0.02; fat 5.86*%* ± 0.15*%* (w/w); protein 4.95*%* ± 0.03*%* (w/w); fat/protein ratio 1.19 ± 0.02; casein 4.04*%* ± 0.02*%* (w/w); lactose 4.62*%* ± 0.04*%* (w/w); urea 40.40 ± 0.02 mg/dL; TSs 16.40*%* ± 0.10*%* (w/w); SCC 1033 ± 151 × 103 cells/mL.

### 3.3. Effect of SMB Powder on Yoghurt Characteristics

#### 3.3.1. Acidification Curves

Despite starting from the same pH, the YSMB formulation demonstrates significantly slower pH reduction kinetics than YC, as evidenced by the kinetic constant *k*_pH_ obtained (Table 2). This results in a delay of approximately 1.5 hours for YSMB in reaching the target pH of 4.67, compared to YC.

**Table 2:** Effect of SMB powder incorporation on initial pH, time to reach pH 4.67, and *k* process rate of yoghurt samples.

Sample	Initial pH	Time pH 4.67 (h, min)	*k*_pH_ × (10^−2^)/h	*R* ^2^
YC	^a^6.38 ± 0.07	^b^4 h 40 min ± 28 min	^a^8.10 ± 0.90	0.91
YSMB	^a^6.29 ± 0.07	^a^6 h 09 min ± 30 min	^b^5.82 ± 0.53	0.93

*Note:* Values represent the mean ± standard deviation (*n* = 3). Different superscripts within each column indicate significant differences following one‐way analysis of variance (ANOVA) and Tukey HSD test (*p* < 0.05).

Abbreviation: *k*_pH_, kinetic constant.

The main parameters calculated from the first derivative of the acidification curves are reported in Table 3.

**Table 3:** Main parameters obtained from the analysis of the first derivative of the acidification curves of yoghurt samples.

Sample	Vm (pH mU/min)	Tm (min)	pHm (pH units)	T_50_ (min)	H_50_ (pH units)
YC	^a^21.7 ± 0.6	^a^163 ± 15	^a^5.6 ± 0.1	^b^37 ± 6	^b^0.65 ± 0.07
YSMB	^b^12.3 ± 0.6	^a^193 ± 12	^a^5.7 ± 0.1	^a^93 ± 6	^a^0.89 ± 0.04

*Note:* Values represent the mean ± standard deviation (*n* = 3). Different superscripts within each column indicate significant differences following one‐way analysis of variance (ANOVA) and Tukey HSD test (*p* < 0.05).

Abbreviations: pH_50_, range of the pH during T_50_; pHm, pH corresponding to Vm; T_50_, time during which the rate of pH reduction is higher than Vm/2; Tm, time corresponding to Vm; Vm, maximum acidification rate.

**Figure 3:**
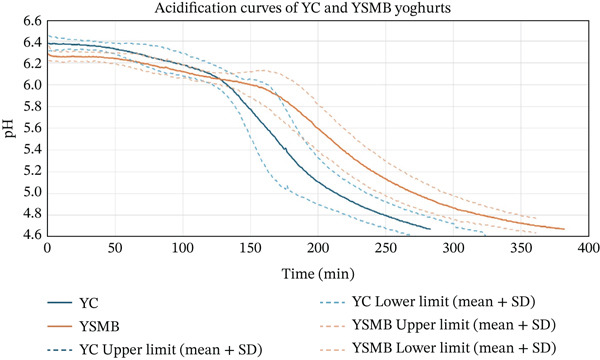
Acidification curves (pH vs. time) of YC and YSMB yoghurts. Data are expressed as mean ± standard deviation (*n* = 3). Solid lines represent mean pH values, whereas dashed lines show the upper and lower limits corresponding to mean ± SD. Fermentation was monitored until samples reached the target pH of 4.67.

The results demonstrate that YSMB exhibits a significantly lower Vm compared to YC (approximately 9 pH mU/min). YSMB requires approximately 30 min more than YC to reach Vm, although this variation does not attain statistical significance. The two yoghurt types reach the same pH at their respective maximum acidification rates. Conversely, YSMB exhibited an acidification rate exceeding Vm/2 for a longer duration than YC, resulting in a pH jump that was greater than that observed in the control. This behaviour may suggest a partial inhibitory effect of the bioactive compounds present in the SMB powder on the starter culture, initially slowing the acidification but allowing a more prolonged and cumulative acidification over time (Figure 3). This finding further supports the marked resistance of lactic acid bacteria (LAB) to polyphenols compared with other bacteria, including pathogenic microorganisms [69, 70]. Furthermore, several studies have reported that plant‐derived polyphenolic ingredients are compatible with yoghurt fermentation and may even support LAB viability [71, 72].

#### 3.3.2. Proximal Composition of YC and YSMB Yoghurts

As shown in Table 4, the proximal composition of the YC and YSMB samples is reported. As can be seen, there were significant differences in TSs, protein and ash among the fortified yoghurt and the control. The YSMB sample was characterised by a higher TS and protein content and a significantly higher ash content compared to the YC sample. These results can be ascribed to the fortification of YSMB with the SMBs powder. No differences were detected in the lactose content or fat analysis. As expected, no significant differences between the fortified yoghurt and the control were observed for the proteolysis indices—the ratios of soluble nitrogen to total nitrogen (SN/TN%), trichloroacetic acid–soluble nitrogen to total nitrogen (TCA‐SN/TN%) and phosphotungstic acid–soluble nitrogen to total nitrogen (PTA‐SN/TN%).

**Table 4:** Proximal composition of YC and YSMB yoghurt samples.

Sample	TS (g/100 g)	Fat (g/100 g)	Protein (g/100 g)	SN/TN (%)	SN‐TCA/TN (%)	SN‐PTA/TN (%)	Ash (g/100 g)	Lactose (g/100 g)
YC	^b^16.14 ± 0.05	^a^5.58 ± 0.20	^b^4.99 ± 0.02	^a^6.57 ± 1.03	^a^5.60 ± 1.41	^a^3.98 ± 0.86	^a^0.59 ± 0.14	^a^3.31 ± 0.50
YSMB	^a^17.15 ± 0.09	^a^5.56 ± 0.42	^a^5.08 ± 0.05	^a^7.35 ± 1.42	^a^6.88 ± 1.73	^a^5.14 ± 0.73	^b^0.72 ± 0.10	^a^3.33 ± 0.95

*Note:* Values represent the mean ± standard deviation (*n* = 6). Different superscripts within each column indicate significant differences following one‐way analysis of variance (ANOVA) and Tukey HSD test (*p* < 0.05).

Abbreviations: SN/TN, soluble nitrogen to total nitrogen ratio; SN‐PTA/TN (%), phosphotungstic acid–soluble nitrogen to total nitrogen ratio; SN‐TCA/TN, trichloroacetic acid–soluble nitrogen to total nitrogen ratio; TS, total solid.

#### 3.3.3. Microbiological Analyses of YC and YSMB Yoghurt Samples

**Figure 4:**
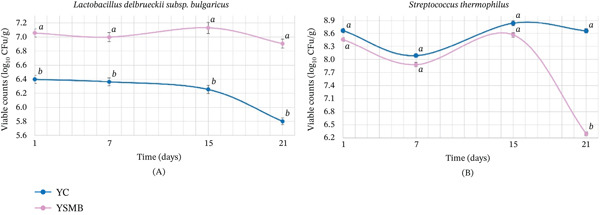
Viable counts of (A) *Lactobacillus delbrueckii* subsp. *bulgaricus* and (B) *Streptococcus thermophilus* of YC and YSMB yoghurt samples during storage at 4°C over 21 days. Data are expressed as mean log_10_ CFU/g ± standard deviation (*n* = 6). Different letters at the same sampling time indicate statistically significant differences (*p* < 0.05).

The presence of viable LAB in fermented milk is a pivotal indicator of product quality. According to Italian regulations [73], yoghurt must contain a minimum of 10^7^ colony‐forming units (CFU) per gram of *S. thermophilus* and *L. delbrueckii* subsp. *bulgaricus* at the time of consumption. Moreover, it is essential that the combined viable count of these two species remains at a level of 10^6^ CFU/g or above until the expiry date indicated on the product packaging. In the present study, the total LAB count was found to be consistently higher than the legal threshold. This finding confirms the microbiological stability of the product and its compliance with regulatory standards [74, 75]. In the YC and YSMB samples, *S. thermophilus* and *L. delbrueckii* successfully grew in ovine milk, completing fermentation (Figure 4). During the storage period (up to 21 days), an increase in the cocci‐to‐bacilli ratio was observed, coinciding with a significant decrease in lactobacilli counts (*p* < 0.001). This decline is likely attributable to the accumulation of lactic acid, which has been shown to create a harsh, inhibitory environment for LAB (Figure 4A). The count of *S. thermophilus* remained constant during the storage period under observation but exhibited a slight decrease, though not to a significant extent, at the time of the final sampling (21 days) (Figure 4B). No significant differences were observed between the YC and YSMB samples. The preponderance of cocci counts over bacilli counts in all samples has been extensively documented, and this phenomenon is commonly linked to reduced postacidification [65] and enhanced flavour development [66]. It was generally observed that populations of *L. bulgaricus* and *S. thermophilus* exhibited an initial increase, followed by a gradual decline during storage [65]. It is noteworthy that in the present study, this decline began as early as Day 7, with significant differences between the YC and YSMB samples in *L. delbrueckii* counts (*p* < 0.001). Throughout the storage period, the YSMB sample exhibited elevated levels of *L. delbrueckii* in comparison to the YC sample. As demonstrated by the data, a significant decrease is observed after 21 days of storage for both samples. The enhanced survival observed in YSMB samples may be attributed to the presence of SMBs, whose polyphenolic compounds exert AOA and may protect LAB under stress conditions [67, 68, 73, 74]. Moreover, lactobacilli have been shown to have the ability to metabolise phenolic compounds of a plant nature through enzymatic systems such as esterases, tannase, decarboxylases and reductases. This metabolic pathway leads to the production of compounds that may enhance bacterial survival under the stressful conditions encountered in yoghurt, including low pH and other growth‐limiting factors. Yoghurt starter LAB are well adapted to fermented dairy environments and can rapidly colonise the milk matrix. Their rapid growth and acidification may counteract the development of undesirable microorganisms, including pathogens and spoilage bacteria. These findings indicate that the levels of polyphenols present in the samples were compatible with LAB viability, and that interactions between phenolic compounds and milk proteins may have further mitigated any inhibitory effects [76]. However, several studies have reported that phenolic compounds, under certain conditions, may exert antimicrobial effects depending on their concentration and molecular structure, potentially inhibiting the growth of LAB [77]. Conversely, other studies have shown that the incorporation of plant materials such as apple pulp, grape pomace or bilberry berries into dairy matrices does not adversely affect LAB growth or fermentation performance [78, 79]. No pathogens or spoilage microorganisms were detected in the YC and YSMB samples during storage, thereby emphasising the safety of the product. This finding indicates that the incorporation of SMB not only preserves the viability of LAB but may also enhance the sensory and functional properties of the final product.

#### 3.3.4. WHC and Syneresis of YC and YSMB Yoghurt Samples

As shown in Table 5 a significant difference can be observed in both the WHC and syneresis between YC and YSMB, with the latter parameter showing a notable increase in the yoghurt fortified with SMB compared to the control. It has been observed that SMB powder demonstrates a similar behaviour to that exhibited by grape extract [80]. However, the soluble fibre content of SMB powder could act as a stabiliser, forming a three‐dimensional network with water. Nevertheless, in this case, the increase in syneresis was found to be particularly pronounced. Furthermore, the addition of polyphenols can result in an augmentation of pore size in the gel matrix, thereby increasing syneresis [80] and concurrently reducing gel strength. As reported by Han et al. [81, 82], the interaction of polyphenols with proteins has been demonstrated to affect the milk protein network. Indeed, it has been demonstrated that the gel‐like consistency of yoghurt is attributable to hydrophobic and electrostatic interactions between proteins. Polyphenols have been demonstrated to reduce these interactions, resulting in a decrease in WHC and, consequently, an increase in syneresis during storage. However, it is important to emphasise that not all the matrices that contain polyphenols demonstrate equivalent behaviour. As Oliveira et al. [83] discovered, the addition of strawberry preparation to yoghurt resulted in a decrease in syneresis, owing to an enhancement in the stability of milk caseins when bound to polyphenols.

**Table 5:** WHC, syneresis, TPC, AOA and colour parameters of YC and YSMB yoghurt samples.

Sample	WHC (%)	Syneresis (%)	TPC (mg GAE/100 g)	AOA (*μ*mol TE/g)	L^*^	a^*^	b^*^	*Δ*E^*^
YC	^a^91.15 ± 1.22	^b^2.85 ± 0.48	^b^1.35 ± 0.05	^b^0.41 ± 0.13	^a^66.06 ± 0.43	^b^−3.18 ± 0.07	^a^5.50 ± 0.16	10.93
YSMB	^b^85.41 ± 2.51	^a^8.11 ± 1.98	^a^8.46 ± 0.33	^a^1.76 ± 0.13	^b^55.33 ± 0.23	^a^1.37 ± 0.09	^b^4.75 ± 0.26	

*Note:* Values represent the mean ± standard deviation (*n* = 9). Different superscripts within each column indicate significant differences following one‐way analysis of variance (ANOVA) and Tukey HSD test (*p* < 0.05).

Abbreviations: AOA, antioxidant activity; TPC, total phenolic content; WHC, water‐holding capacity.

#### 3.3.5. TPC and AOA of YC and YSMB Yoghurts Samples

TPC in YSMB was found to be significantly higher than in the control group, as had been hypothesised (Table 5). The incorporation of 1% of SMB powder resulted in a significant increase in the content of TPC and, consequently, AOA in the YSMB sample compared with the YC sample.

However, the total polyphenol content of the YSMB sample was lower than expected. As reported by Du et al. [47, 84], the incorporation of polyphenols in yoghurt is dose‐dependent, and their presence gradually increases during storage. Getsemani et al. [85] further reported that the increase in phenolic compounds in yoghurts enriched with non‐dairy ingredients can be attributed to the presence of naturally occurring bioactive compounds, such as flavonoids (quercetin and kaempferol) and hydroxycinnamic acids (gallic, caffeic and chlorogenic acids). The observed changes during cold storage may also be related to the release of phenolic compounds previously bound to polysaccharides and proteins. As widely reported in the literature [47, 86, 87], polyphenols are known to interact with milk proteins through hydrogen bonding, hydrophobic interactions and covalent bonds [87], leading to the formation of both soluble and insoluble complexes. In particular, the strong binding between phenolic compounds and caseins, especially under acidic pH conditions, may reduce their extractability and thus their apparent recovery [86]. Such low recovery of total polyphenols has also been reported in yoghurts fortified with strawberry preparations, tomato pomace and pomegranate juice [76]. However, it is important to note that the evolution of phenolic content during storage is not always consistent across studies. For instance, Dimitrellou et al. [6] observed a decrease in total phenolic compounds during storage, particularly in yoghurts supplemented with fruit juices, suggesting that certain extracts may be more susceptible to degradation under refrigerated conditions.

As expected, the AOA of fortified yoghurts was found to be higher than that of the control (Table 5). Moreover, a poor correlation was observed in the literature between the antioxidant capacity and TPC in acid gels. This phenomenon is likely attributable to the occurrence of acidification conditions, which result in enhanced interaction between whey proteins present in the upper layer of the mixture and polyphenols [88]. However, the interaction between proteins and polyphenols has been demonstrated to exert a protective effect on polyphenols during the process of digestion [86]. It has been demonstrated that this interaction leads to an enhancement in the bioaccessibility and bioavailability of polyphenols, in addition to an increase in AOA.

#### 3.3.6. Colour Analysis YC and YSMB Yoghurts Samples

A substantial decrease in L^*^ was evident in YSMB in comparison to YC, which can be attributed to the presence of the by‐products incorporated (Table 5). The YSMB sample exhibited an increase in the a^*^ coordinate, which can be attributed to the anthocyanin content of the exhausted myrtle berries. The overall colour difference (*Δ* *E* ^∗^ = 10.93) indicates a visually perceptible change because a *Δ*E^*^ value greater than 3 is generally distinguishable by the human eye. This finding is consistent with the literature on mulberry pomace [47].

#### 3.3.7. SEM Analysis on YC and YSMB Yoghurt Samples

**Figure 5:**
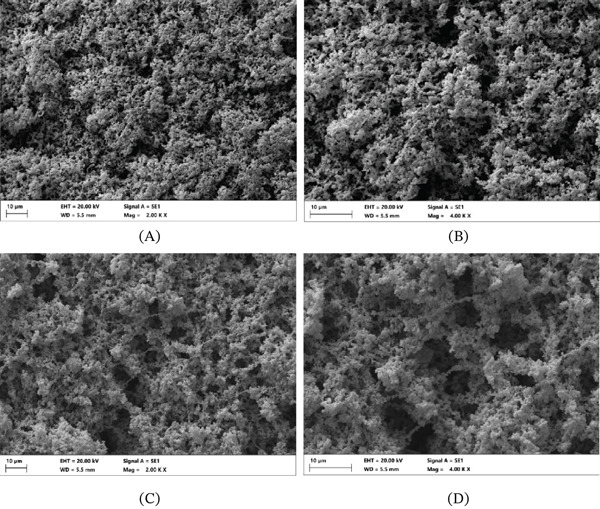
SEM images of yoghurts, at different scale bars and magnifications. YC: (A) 10 *μ*m–2.00 K x and (B) 10 *μ*m–4.00 K x. YSMB: (C) 10 *μ*m–2.00 K x and (D) 10 *μ*m–4.00 K x.

In order to elucidate the influence of fortification with SMB powder, the microstructure of YC and YSMB was evaluated through SEM (Figure 5). As is evident from the figure, YC displayed a dense three‐dimensional network formed by aggregates of casein micelles, which confer a grainy aspect to the product, with some void areas. The fortification of the yoghurt with SMB powder has been shown to influence its microstructure, resulting in a disruption of the protein network and the creation of a more open network. This phenomenon is likely attributable to the binding between the polyphenols and milk proteins through hydrophobic interactions, which results in the weakening of the microstructure and consequent effect on all the textural and rheological parameters [13, 89].

#### 3.3.8. Texture Analysis of YC and YSMB Yoghurt Samples

The texture of yoghurt is widely considered to be one of the most significant quality attributes. The gel structure is the result of two processes: the aggregation of casein triggered by pH reduction and the formation of disulfide bonds between denatured whey proteins, resulting from milk heat treatment and k‐caseins. Yoghurt fortification with SMB powder significantly decreased firmness and stickiness (Table 6). This reduction may be explained by the water‐retention capacity of the by‐products, which increases gel softness by entrapping water within the matrix [47]. Additionally, Osorio‐Arias et al. [13] suggested that phenolic‐rich by‐products can weaken yoghurt firmness by disrupting the protein network established between denatured whey proteins and caseins, which plays a crucial role in maintaining the gel structure.

**Table 6:** Textural parameters of YC and YSMB yoghurt samples.

Sample	Firmness (N)	Work of shear (N·s)	Stickiness (N)	Work of adhesion (N·s)
YC	^a^0.63 ± 0.02	^a^8.94 ± 0.31	^b^−0.23 ± 0.01	^b^−4.60 ± 0.37
YSMB	^b^0.30 ± 0.01	^b^3.85 ± 0.10	^a^−0.09 ± 0.01	^a^−1.63 ± 0.24

*Note:* Values represent the mean ± standard deviation (*n* = 9). Different superscripts within each column indicate significant differences following one‐way analysis of variance (ANOVA) and Tukey HSD test (*p* < 0.05).

#### 3.3.9. Rheological Analyses of YC and YSMB Yoghurt Samples

The rheological data can be found in Tables 7 and 8. The steady shear rheological analysis revealed that all the samples exhibited shear‐thinning fluid behaviour (*n* < 1), where the viscosity decreased at an increasing shear rate, as evidenced by the values of the flow behaviour index. The YSMB yoghurt exhibited a significant decrease in consistency index, apparent viscosity and Casson yield stress, in comparison to the YC. It can be hypothesised that this phenomenon may be attributable to the diminution of the capacity of milk proteins to bind water, consequent to the incorporation of by‐product powders. This, in turn, results in a reduction of the three‐dimensional structure of the acid gel [90]. This finding is consistent with the results reported in numerous publications in this subject area [13, 91]. The G′, G″ and *η* values of YSMB were found to be significantly lower than those of the YC sample, revealing a significantly more fluid consistency for the fortified yoghurt [61]. Indeed, as can be observed, G′ was found to be higher than G″, thus conferring to the YSMB sample a structure of a weak elastic gel and solid‐like behaviour [61, 63]. These findings confirmed that the incorporation of SMB powder, a notable source of polyphenols, may diminish the interactions among proteins, thus reducing the gel strength [81, 82].

**Table 7:** Steady shear rheological parameters of YC and YSMB yoghurt samples.

Sample	Flow behaviour index (*n*)	Consistency index (K) (Pa·sⁿ)	Apparent viscosity *η*_a_,₅₀ (Pa·s)	Casson yield stress *σ*_ac_ (Pa)
YC	^b^−0.87 ± 0.01	^a^24.91 ± 2.41	^a^0.91 ± 0.04	^a^1.87 ± 0.02
YSMB	^a^−0.73 ± 0.01	^b^7.11 ± 0.68	^b^0.51 ± 0.01	^b^1.67 ± 0.03

*Note:* Values represent the mean ± standard deviation (*n* = 9). Different superscripts within each column indicate significant differences following one‐way analysis of variance (ANOVA) and Tukey HSD test (*p* < 0.05).

**Table 8:** Frequency sweep rheological test on YC and YSMB yoghurt samples.

Sample	Storage modulus (G′)	Loss modulus (G″)	Complex viscosity *η* ^*^	Tan *δ*
YC	^a^451.03 ± 85.11	^a^113.04 ± 26.46	^a^63.48 ± 12.12	^b^0.25 ± 0.02
YSMB	^b^162.74 ± 8.97	^b^48.97 ± 2.74	^b^23.20 ± 1.26	^a^0.30 ± 0.01

*Note:* Values represent the mean ± standard deviation (*n* = 9). Different superscripts within each column indicate significant differences following one‐way analysis of variance (ANOVA) and Tukey HSD test (*p* < 0.05).

#### 3.3.10. Sensory Evaluation of YC and YSMB Yoghurt Samples

**Figure 6:**
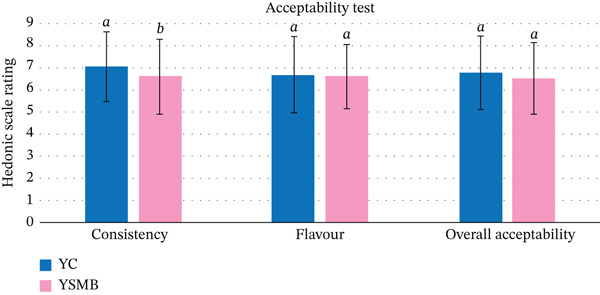
Acceptability test of YC and YSMB samples. Acceptability scores represent the mean ± standard deviation (*n* = 100). Different superscripts within each column indicate significant differences following one‐way analysis of variance (ANOVA) (*p* < 0.05).

Because textural defects such as syneresis and reduced gel firmness are known to affect sensory acceptance [92], consumer perception was investigated through an acceptability test. The results showed a statistically significant difference in the consistency attribute between YC and YSMB (*p* < 0.05), with YSMB being perceived as less consistent than the control sample (Figure 6). These findings are in agreement with the instrumental texture analyses, which demonstrated lower firmness and adhesiveness in YSMB in comparison to the YC control. Nevertheless, despite the differences in textural properties, no significant differences were detected between the two samples in terms of flavour and overall liking, indicating that the reduced consistency of YSMB did not compromise consumers′ overall acceptance of the product.

## 4. Conclusions

The present study revealed the potential of utilising powder derived from exhausted myrtle berries to produce a yoghurt with increased nutritional value, attributable to the contribution of polyphenols and enhanced antioxidant capacity. However, this by‐product was found to cause some imperfections in the yoghurt, including increased syneresis and reduced gel strength. This outcome was attributed to the disruption of the protein network caused by the interaction between polyphenols and milk proteins. This interaction resulted in weakened microstructure and affected all the textural and rheological parameters, as evidenced by SEM analysis. The study revealed that SMB powder is a safe ingredient with no adverse effect on starter culture survival. However, it can be asserted that the implementation of an optimisation strategy is imperative to enhance the rheological and textural characteristics of yoghurt. Moreover, the absence of significant differences in the flavour attribute suggests that the addition of SMB to yoghurt prior to pasteurisation did not adversely affect sensory quality, despite the high polyphenol content, which can lead to the presence of bitterness and astringency. This hypothesis is corroborated by the findings of the overall acceptance attribute, which demonstrate no statistically significant differences between the fortified product and its control. Nevertheless, optimisation of the formulation is required to improve the rheological and textural properties of the yoghurt. This could be achieved by the incorporation of hydrocolloids or polysaccharides, which must ensure that no adverse impact is exerted on the sensory characteristics of the product. Moreover, further research is necessary to evaluate the product′s stability and quality over its shelf life. It can be concluded that SMB powder can be regarded as a sustainable functional ingredient in dairy applications.

## Author Contributions

Angela Carboni: research execution, methodology development, formal analysis and writing—original draft preparation. Roberto Cabizza: research execution, methodology development, formal analysis and writing—original draft preparation. Pietro Paolo Urgeghe: conceptualization, supervision, writing—original draft preparation and writing—review and editing. Francesco Fancello: formal analysis and writing—original draft. Massimo Pes: methodology development, writing—original draft and writing—review and editing. Alessio Silvio Dedola: formal analysis. Severino Zara: writing—review and editing. Alessandra Del Caro: conceptualization, data collection, writing—original draft and writing—review and editing.

## Funding

No funding was received for this manuscript.

## Disclosure

All authors have read and approved the final version of the manuscript, consented to the submission for publication and agreed to be fully accountable for all aspects of the research presented.

## Ethics Statement

Ethical review and approval were waived for this study because the participation was voluntary. Individuals with lactose intolerance or known allergies to myrtle were excluded from participation. All data were anonymous. Informed consent was obtained from all subjects involved in the study in accordance with current regulations and legislation relevant to food science, including the General Data Protection Regulation (GDPR, EU Regulation 2016/679). As the study did not involve vulnerable populations and was classified as low‐risk, ethical committee approval was deemed unnecessary, in line with the European Commission′s ethics review procedures and IFST guidelines.

## Conflicts of Interest

The authors declare no conflicts of interest.

## Data Availability

The data that support the findings of this study are available from the corresponding author upon reasonable request.
